# Injections of Linseed Oil for the Cure of Chronic Cystitis

**Published:** 1879-09

**Authors:** 


Imjections of Linseed Oil for the Cure of Chronic Cystitis.—A man, aged twenty-nine years, entered the hospital December 23, suffering from cystitis of six months' standing. Micturition occurred every hour both day aud night. The urine contained a large amount of mucus and pus. The ordinary remedies were used without benefit, and finally Dr. Howe proposed to distend the bladder and keep it so as long as possible. The agent he used was linseed oil; eight ounces were used at each daily injection. After the treatment had been continued for a week, the cystitis improved. The pus and mucus disappeared. Micturition occurred only six times in twenty-four hours, and was unattended with pain.

Another patient, aged forty-nine years, was admitted with cystitis of three months' standing. Urine contained both pus and mucus. Micturition was painful, and occurred eighteen times a day. The injections of linseed oil were used as in the previous case. After eight days the pain abated, and he was able to hold his urine for two hours; but at that time he left the hospital and has not reported since.—*New York Medical Journal.*

